# ﻿A new species of woodlouse (Isopoda, Oniscidea) from the Canarian laurel forest

**DOI:** 10.3897/zookeys.1225.124521

**Published:** 2025-02-05

**Authors:** Raúl Orihuela-Rivero, Carmen Balibrea, Víctor Noguerales, Heriberto López, Pedro Oromí

**Affiliations:** 1 Instituto de Productos Naturales y Agrobiología (IPNA), CSIC, C/Astrofísico Francisco Sánchez 3, 38206 La Laguna, Santa Cruz de Tenerife, Canary Islands, Spain; 2 School of Doctoral and Graduate Studies, University of La Laguna, C/Astrofísico Francisco Sánchez s/n, 38200 La Laguna, Santa Cruz de Tenerife, Canary Islands, Spain; 3 Department of Animal Biology, Edaphology and Geology, University of La Laguna, C/Astrofísico Francisco Sánchez s/n, 38200 La Laguna, Santa Cruz de Tenerife, Canary Islands, Spain

**Keywords:** Canary Islands, Porcellionidae, taxonomy, Tenerife, woodlice

## Abstract

A new species of terrestrial isopod, *Porcellioaguerensis* Orihuela-Rivero, **sp. nov.** of the family Porcellionidae (Oniscidea), is described from the laurel forest of Tenerife, Canary Islands. This new species belongs to the Atlantic group (“*scaber*”) as defined by Vandel due to the structure of the male pleopod 1 and its “primitive” glandular system. Some diag­nostic characters that allow it to be differentiated from other species are revealed, such as (i) the smooth dorsal surface, (ii) the sinuosity of the posterior margin of the first pereonites, (iii) the configuration of the glandular system, and (iv) the structure of the male pleopod 1 exopod. The affinity of *Porcellioaguerensis* Orihuela-Rivero, **sp. nov.** with the morphologically closest members of the genus is discussed, both with continental and insular species, hypothesizing a relationship between the Canarian species of *Porcellio* and the “primitive” continental lineages of the genus. A key of the *Porcellio* species occurring in Tenerife is included. The conservation of *Porcellioaguerensis* Orihuela-Rivero, **sp. nov.** within a scenario of increasing dominance of invasive species is discussed.

## ﻿Introduction

The terrestrial isopod fauna of the Canary Islands remains notoriously understudied. Until the recent publication of the description of *Soteriscusjandiensis* Cifuentes & Prieto, 2023, no new taxon of the suborder Oniscidea had been described for the archipelago for 16 years (Taiti and López 2008). The Canary Islands host a high diversity of terrestrial isopods, which has already been demonstrated in 1991 by Rafael Rodríguez Santana in his detailed but unfortunately unpublished doctoral thesis. The archipelago harbours at least 35 native species, 29 of which are endemic (Gobierno de Canarias 2024). Of these,17 species belong to the genus *Porcellio* Latreille, 1804.

The family Porcellionidae is composed of 19 genera, making it the fourth most diverse family (over 330 species) of Oniscidea (Sfenthourakis and Taiti 2015). The family is characterized by (i) the flagellum of second antenna having two joints, (ii) the absence of conglobational ability (except *Atlandidium* Arcangeli, 1936), and (iii) the presence of monospiracular covered lungs on pleopod 1 and 2 exopods (Schmidt 2003). Despite these shared morphological characters, recent molecular studies question the monophyly of Porcellionidae and the monophyly of several of its constituent genera, such as *Porcellio* and *Porcellionides* Miers, 1877 (Dimitriou et al. 2018).

In the Canary Islands, Porcellionidae is represented by 32 species, of which 22 are endemic, with half of them described in the last 40 years (Dalens 1984; Hoese 1985; Rodríguez Santana 1990; Rodríguez and Vicente 1992a, 1992b, 1992c; Rodríguez and Barrientos 1993; Cifuentes and Prieto 2023). Within this family, *Porcellio* is the genus with the highest species richness, with 22 species representing the halophilic (“*lamellatus*”), North African (“*laevis*”), and Atlantic (“*scaber*”) groups following the group-classification of Vandel (1951, 1956, 1962). This classification, which encompasses several morphological characters and homogeneous distributions, although it is accepted by most experts as a very useful tool to deal with this diverse genus, it still needs to be tested with molecular analysis to rule out that these similarities are due to evolutionary convergence phenomena.

The aim of this study is to describe a new species of the genus *Porcellio* from Tenerife (Canary Islands) and to discuss its similarities and differences with what appear to be closely related species, both insular and continental.

### ﻿Study area

**Figure 1.**
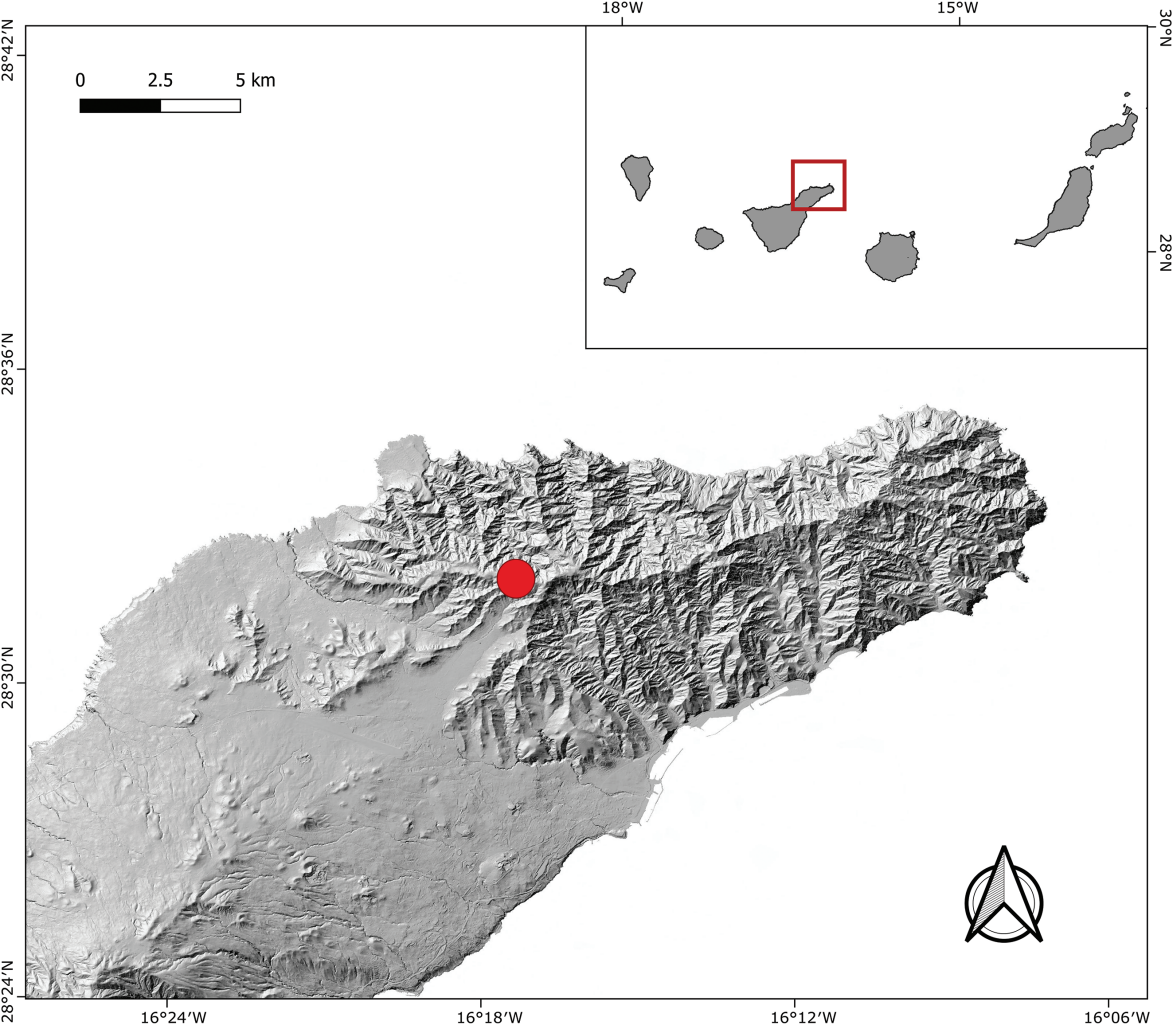
Geographic location of Tenerife within the Canary Islands and sampling sites in Anaga massif (red point).

Tenerife is situated in an almost central position within the archipelago of the Canary Islands (Fig. 1) located off the northwest coast of Africa in the Atlantic Ocean. Like all of the Canary Islands, Tenerife has a volcanic origin, although it is thought to have originally formed from three paleoislands (Roque del Conde, Teno, and Anaga) that later merged as a consequence of more recent volcanic episodes (Carracedo and Pérez-Torrado 2013). Our sampling took place in the Anaga massif (Fig. 1), with an estimated age of 3.9–4.9 million years (My) (Guillou et al. 2004). Its age and high geological stability (Ancochea et al. 1990) have promoted long periods of intense erosion producing landscapes with complex topographies that have stimulated geographical isolation of po­pulations and, ultimately, processes of speciation (Fernández-Palacios et al. 2017; Salces-Castellano et al. 2020). Due to an east-west orientation of the dorsal ridge of the peninsula, and its altitude of up to 1024 m above sea level (a.s.l.), orographic cloud formations are a feature of the massif, supplying up to 1200 mm of water per year by horizontal precipitation (Fernández-Palacios et al. 2017; del Arco and Rodríguez-Delgado 2018). This cloud formation sustains the presence of the laurel forest, a relict arboreal plant formation rich in laurophilous trees comparable to the humid subtropical evergreen rainforests that spread over the Mediterranean during the Palaeogene and Neogene (but see Kondraskov et al. 2015). On spurs and ridges between 600 and 1100 m a.s.l., there is a dense, low to medium-altitude formation dominated by the Macaro­nesian endemic *Ericaplatycodon* (Webb & Berthel.) Rivas-Mart. & al. and other typical laurel forest species such as *Ilexcanariensis* Poir. in Lamarck, *Laurusnovocanariensis* Rivas-Mart., Lousa, Fern. Prieto, E. Días, J.C. Costa & C. Aguiar, and *Morellafaya* (Aiton) Wilbur. Areas of forest with greater exposure to cloud formation and more limited exposure to solar radiation are rich in lichens, mosses and ferns, but there are also periods of desiccation due to near-conti­nuous wind in the upper canopy (del Arco and Rodríguez-Delgado 2018). This is the case of the sampling area, located above 900 m a.s.l. on the western summits of Anaga (Monte de Las Mercedes, Fig. 1), with an average temperature of 15.2 °C and a rainfall of 1050 mm per year (Fernández-Palacios et al. 2017).

## ﻿Methods

The studied specimens were collected within the laurel forest by pitfall traps, with a subset of specimens preserved in absolute ethanol for future mole­cular analysis. Terminology used for the description is based on Vandel (1960). For the examination of anatomical parts, slides for microscopy were prepared with dimethyl hydantoin formaldehyde and temporary preparations in glyce­rol, as appropriate. Preserved specimens were examined with a Zeiss Stemi SV6 stereomicroscope and a Zeiss Axioskop 40 binocular microscope. Images were obtained using the photo stacking technique, attaching a Canon EOS 750D digital camera to these microscopes. Each final image was obtained processing their stack of photographs with the program Zerene Stacker v. 1.04 (Zerene Systems, LLC, Richland, WA), combining them into a single image using pmax and dmap methods. The software Photoshop was used for final retouching. Drawings were made with graphic editor Inkscape v. 1.3. (https://inkscape.org). The type specimens of this new species have been deposited into the Collection of the Institute of Natural Products and Agrobiology, La Laguna, Tenerife (**IPNA**); the Collection of the Department of Animal Biology (Zoology) of the University of La Laguna (**DZUL**); and the Collection of the Museum of Nature and Archaeology of Tenerife (**TFMC**).

## ﻿Results

### ﻿Taxonomy

**Order Isopoda Latreille, 1816**

**Suborder Oniscidea Latreille, 1802**

**Family Porcellionidae Brandt in Brandt & Ratzeburg, 1831**

**Genus *Porcellio* Latreille, 1804**

#### ﻿ Porcellio aguerensis

Taxon classificationAnimaliaIsopodaPorcellionidae

Orihuela-Rivero sp. nov.

021F17F3-F650-5C75-B73B-E70AA8208D10

https://zoobank.org/FF7E5C1A-085B-4785-8387-187AD0D4316B

##### Type material.

***Holotype***: Spain • ♂; Canary Islands, Tenerife, Zapata; 28°32.06'N, 16°17.63'W; 915 m a.s.l.; May 2023; Carmen Balibrea and Víctor Noguerales leg.; pitfall trap; IPNA BC2649. ***Paratypes***: Spain • 1 ♂; Canary Islands, Tenerife, Zapata; 28°31.93'N, 16°17.53'W; 930 m a.s.l.; May 2023; Carmen Balibrea and Víctor Noguerales leg.; pitfall trap; DZUL 36998 • 1 ♀ ovigerous; Canary Islands, Tenerife, Zapata; 28°31.93'N, 16°17.13'W; 960 m a.s.l.; May 2023; Carmen Bali­brea and Víctor Noguerales leg.; pitfall trap; DZUL 36999 • 1 ♂; same collection data as for preceding; BC2650 • 1 ♀ ovigerous; Canary Islands, Tenerife, Zapata; 28°31.93'N, 16°17.19'W; 955 m a.s.l.; May 2023; Carmen Balibrea and Víctor Noguerales leg.; pitfall trap; IPNA BC2651 • 1 ♀; Canary Islands, Tenerife, Zapata; 28°31.99'N, 16°17.55'W; 925 m a.s.l.; May 2023; Carmen Balibrea and Víctor Noguerales leg.; pitfall trap; TFMCEN-2381.

##### Diagnosis.

Teguments smooth; glandular fields associated to lateral margins located in widening of marginal groove of pereonites; d/c coordinate va­lues of noduli laterales with a peak on pereonite 4; pereonites 1–2 with posterior margins slightly sinuous; telson triangular with apex rounded; second antenna not reaching posterior margin of second pereonite; uropods with protopods posterior margin very oblique; exopods narrow. Male pereopod 7 ischium with concave ventral margin and slight distal depression on its rostral face covered with numerous setae; male pleopod 1 exopod with short posterior lobe.

##### Description.

**Figure 2.**
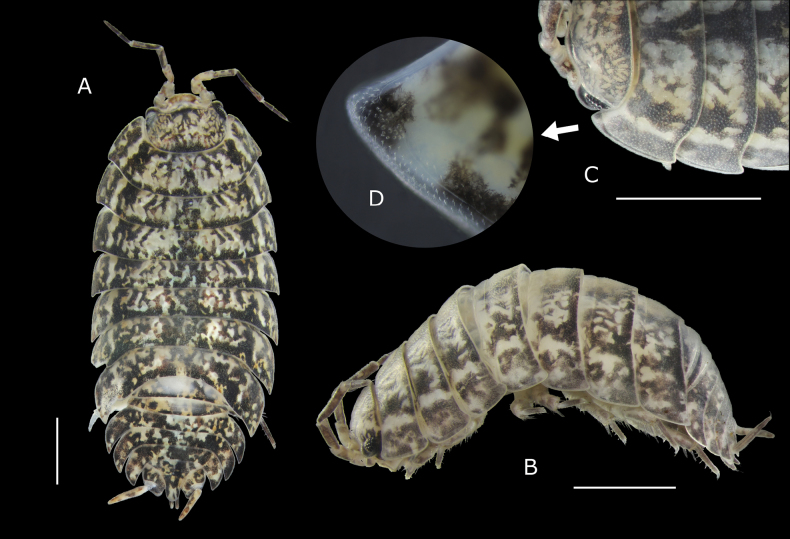
*Porcellioaguerensis* sp. nov. **A** paratype ♀, habitus, dorsal view **B** holotype ♂, habitus, lateral view **C** paratype ♂, details of the first pereonites **D** details of configuration of the glandular field of the first pereonite. Scale bars: 2 mm (**A, B, C**).

**Figure 3.**
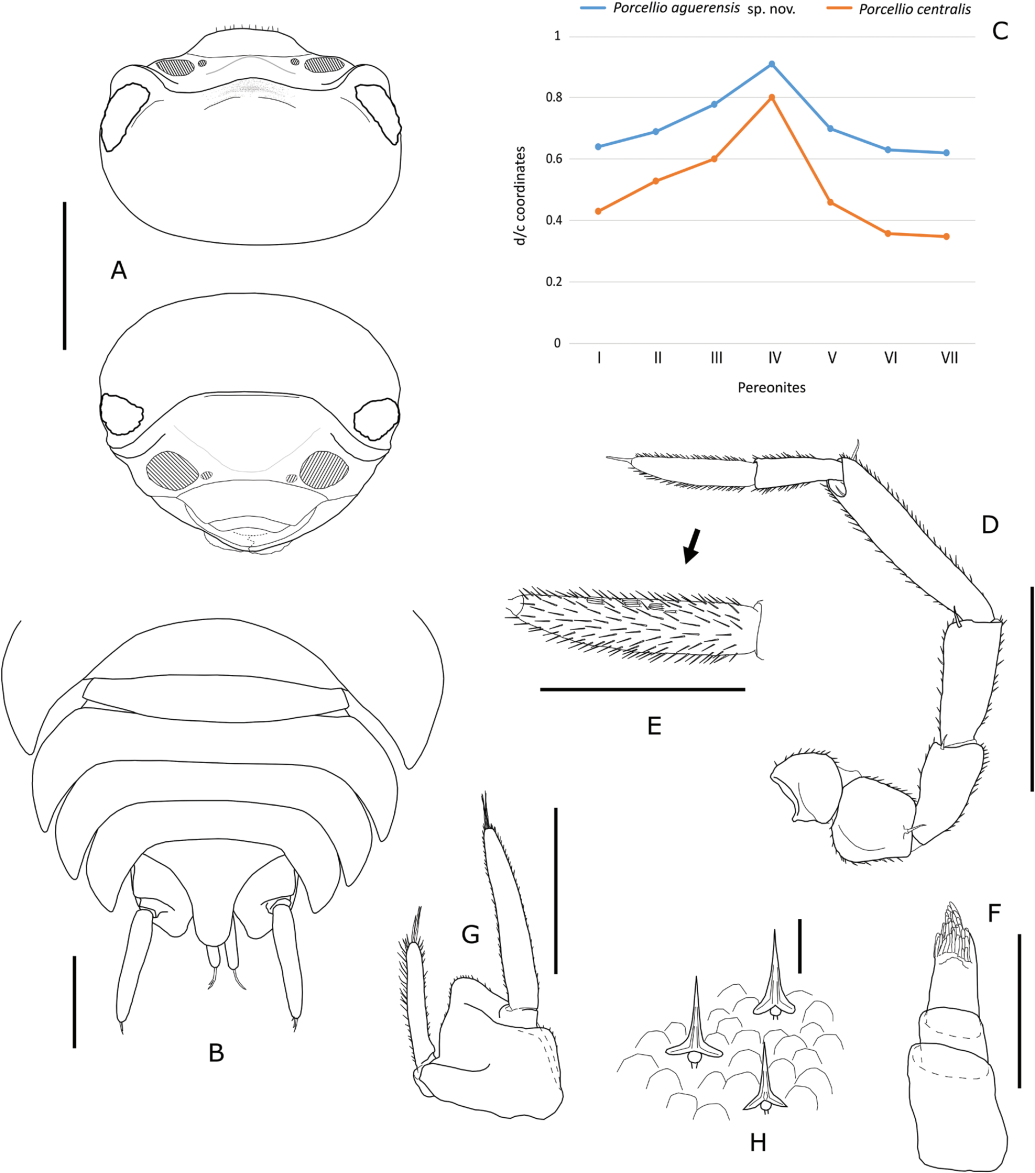
*Porcellioaguerensis* sp. nov. **A** paratype ♀, cephalon, dorsal and frontal view **B** paratype ♀, details of pleon, telson and uropods **C** paratype ♀, noduli laterales d/c coordinates and comparison with *Porcelliocentralis* Vandel, 1954 **D** paratype ♂, second antenna **E** paratype ♂, details of the distal article of antennal flagellum **F** paratype ♂, first antenna **G** paratype ♀, uropod **H** paratype ♀, dorsal cuticular scales and scale-setae. Scale bars: 1 mm (**A, B, D, G**); 0.5 mm (**E**); 0.2 mm (**F**); 0.02 mm (**H**).

**Figure 4.**
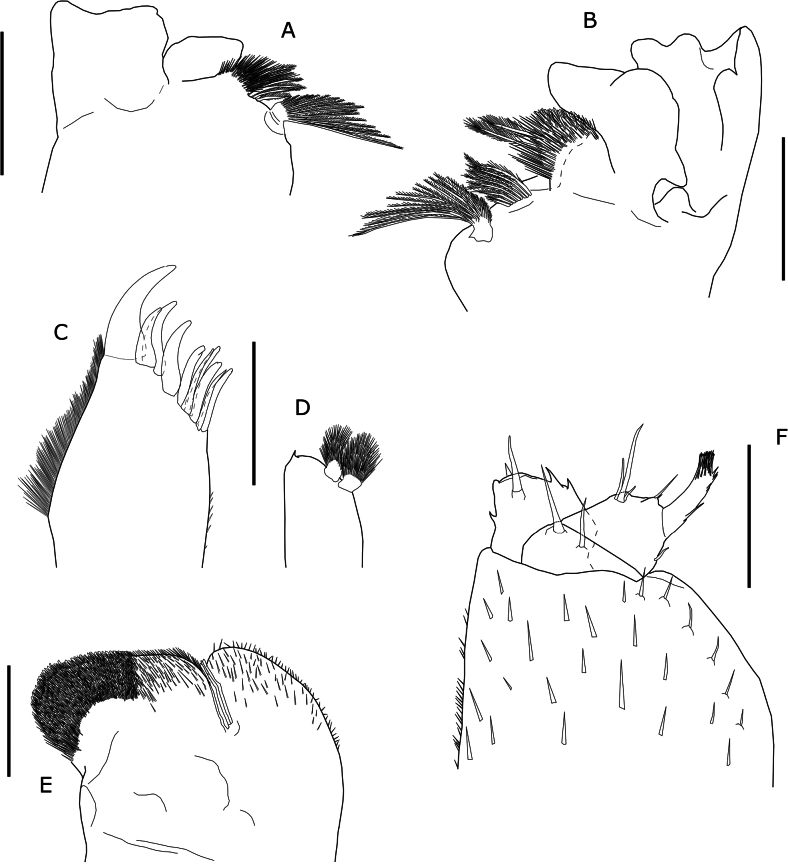
*Porcellioaguerensis* sp. nov., paratype ♂ **A** right mandible **B** left mandible **C** maxillule, external branch **D** maxillule, internal branch **E** maxilla **F** maxilliped. Scale bars: 0.2 mm (**A, B, C, D, F**); 0.1 mm (**E**).

Maximum length: male 8.5 mm, female 9.8 mm. Body strongly convex with epimera oriented obliquely, ovoid (Fig. 2A, B); outline between pereon and pleon continuous. Colour (Fig. 2A–C) mottled brown with irregular broad dark midline reaching posterior margin of cephalon; pereonites 4–7 usually with pairs of paramedian diffuse yellow spots; pigmented epimera, presenting small depigmented area around noduli laterales and band of depigmentation at base of epimera; pereopods and pleopods pigmented; dark maxilliped; uropod exopods often reddish or brown. Cephalon, pleon and telson smooth. Dorsal surface covered with large Y-shaped scale-setae (Fig. 3H) and imbri­cated rounded scales. Pereonites 1–7 epimera with distinct groove along entire late­ral margin with one widening concentrating glandular pores ranging from 6 to 20, number of pores gradually decreasing towards posterior pereonites; widening located at anterior or in middle portion of epimera, in the first pereonite at anterior corner of epimeron (Fig. 2D). One line of large noduli laterales per side on pereonites 1–7, far from lateral margins, with high d/c ratio as in Fig. 3C. Cepha­lon (Fig. 3A) without supra-antennal line; frontal-line arches slightly forming rounded small median lobe; lateral lobes rounded or slightly quadrangular; eyes with 24–28 ommatidia. Pereonites 1–2 with posterior margins slightly sinuous (Fig. 2C); 3–6 straight, posterior corners slightly directed backwards; 7, regularly concave. Pleonites 3–5 with well-developed epimera; posterior corners bending backwards; pleonite 5 epimera not surpassing uropod protopods or telson apex (Fig. 3B). Telson (Fig. 3B) wider than long, triangular, with concave margins, sides of distal part of some specimens almost parallel; apex rounded. First antenna (Fig. 3F) tri-articulated; basal and distal article subequal in length; distal article with tuft of about 30 subapical aesthetascs. Second antenna (Fig. 3D, E) not reaching posterior margin of second pereonite when extended backwards; finely setose; flagellum bi-articulated, slightly shorter than fifth article of peduncle; second article about 1.5 times longer than first, bearing several rows of aesthetascs (Fig. 3E). Mandibles with dichotomized molar penicil; left mandible (Fig. 4B) with 2 + 9 penicils; right mandible (Fig. 4A) with 1 + 4 penicils. Maxillule external branch (Fig. 4C) with 4 + 6 teeth, four of them subapically cleft; internal branch (Fig. 4D) with two thick penicils and one short sharp posterior corner. Maxilla inner lobe quadrangular (Fig. 4E), wider than outer lobe, apex covered fine and thick setae; outer lobe (Fig. 4E) with three long incurved setae on margin between lobes; covered with thin setae. Maxilliped (Fig. 4F) palp with two strong setae on first article, distal article with one tuft of setae; endite quadrangular, with one strong seta and four triangular teeth, three on distal margin and one on lateral margin. Pereopods short and stout. Pleopod 1 and 2 exopods with monospiracular covered lungs with indented outer margin. Uropodal protopods (Fig. 3G) with ribbed outer margin and very oblique posterior margin, with its inner end almost reaching tip of telson (Fig. 3B); endopod and exopod (Fig. 3G) inserted at different level; exopods narrow, styliform, about 1.5 times longer than endopods; endopods surpassing telson apex (Fig. 3B).

**Figure 5.**
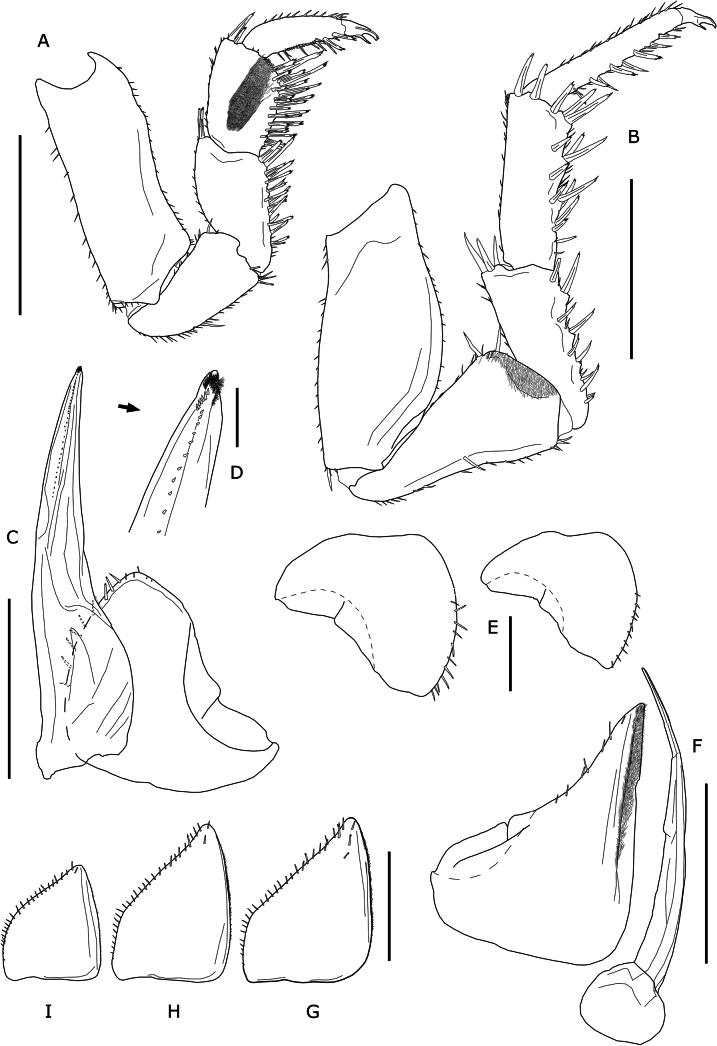
*Porcellioaguerensis* sp. nov., paratype ♂ **A** first male pereopod **B** seventh male pereopod **C** first male pleopod, ventral view **D** distal portion of the first male pleopod endopod, ventral view **E** variation of the first male pleopod, frontal view **F** second male pleopod, frontal view **G** third male pleopod, frontal view **H** fourth male pleopod, frontal view **I** fifth male pleopod, frontal view. Scale bars: 1 mm (**A, B, C, F, G, H, I**); 0.5 mm (**E**); 0.1 mm (**D**).

**Male**: Pereopod 1 (Fig. 5A) merus and carpus with strong setae on sternal margin, more numerous than in female. Pereopod 2–3, also with these modifications, but less noticeable. Pereopod 7 (Fig. 5B) ischium with slightly concave ventral margin and slight distal depression on its rostral face covered with numerous setae. Pleopod 1 exopod (Fig. 5C, E) with short posterior lobe ending in rounded apex or with small protruding tip (Fig. 5E), inner margin with strong setae; endopod (Fig. 5C) about twice as long as exopod, with straight or slightly curved distal margin, apex with row of spines (Fig. 5D). Pleopod 2 (Fig. 5F) exopod triangular, with straight inner margin; distal part of outer margin with some setae; endopod styliform, about 1.3 times longer than exopod. Pleopods 3–5 exopods (Fig. 5G–I) triangular.

##### Etymology.

The species name is derived from “Aguere”, the aboriginal term of the geographic area where currently the municipality of La Laguna is situa­ted and where the species was found.

##### Ecology.

Epigean species. *Porcellioaguerensis* Orihuela-Rivero, sp. nov. has only been recorded so far from the higher altitude laurel-forest areas of the western sector of Anaga, appearing only occasionally in pitfall traps. This species is associated with *Ericaplatycodon* communities, although some traps were also positioned in more humid laurel forest characterized by the presence of *Laurusnovocanariensis*. Several co-occurring Canary Island endemics were also sampled: very abundantly *Porcellioanagae* Hoese, 1985 (family Porcellio­nidae), and more rarely *Ctenorilloausseli* (Dollfus, 1893) (family Armadillidae). *Armadillidiumvulgare* (Latreille, 1804), an invasive Mediterranean species of the family Armadillidiidae (Arndt and Mattern 2005) was also commonly sampled together with *P.aguerensis* Orihuela-Rivero, sp. nov.

### ﻿Identification key of the *Porcellio* species from Tenerife (Canary Islands)

**Table:** 

1	Male pleopod 1 exopod with elongated posterior lobe. Glandular fields not associated with the margin of the epimera	**2**
–	Male pleopod 1 exopod with short posterior lobe. Glandular fields, when present, associated with the margin of the epimera	**3**
2	Dorsal surface without or with some flattened granules; scales absent except on the anterior corner of the tergites	***P.laevis***
–	Dorsal surface with conspicuous, small, rounded granules, especially remarkable on cephalon; scales present on almost the entire surface	***P.alluaudi***
3	Dorsal surface without granules	**4**
–	Dorsal surface with granules	**5**
4	Epimera with few pores distributed in an inconspicuous marginal groove. Male pereopod 7 without noticeable sexual dimorphism. Troglobitic species	***P.martini***
–	Epimera with pores arranged in a glandular field located in a widening of the marginal groove. Male pereopod 7 ischium with concave ventral margin. Epigean species	***P.aguerensis* sp. nov.**
5	Glandular field in a marginal groove occupying all or almost the entire length of the lateral margin of the epimeron	**6**
–	Glandular field located in a limited semi-elliptical region associated to the lateral margin of the epimeron	**8**
6	Uropod endopods not surpassing the telson apex. Noduli laterales in the 4^th^ pleonite with d/c coordinate value > 1	***P.medinae***
–	Uropod endopods surpassing the telson apex. Noduli laterales in the 4^th^ pleonite with d/c coordinate value < 0.85	**7**
7	Rounded telson tip. Male pleopod 1 exopod with respiratory field deeply indented. Male pereopod 7 ischium with concave ventral margin	***P.anagae***
–	Acute telson tip. Male pleopod 1 exopod with respiratory field not inden­ted. Male pereopod 7 ischium without noticeable sexual dimorphism	***P.septentrionalis*** ^1^
8	Glandular fields present only in the first 4 pereonites. Cephalon with large semicircular median lobe	***P.canariensis***
–	Glandular fields present in all pereonites. Cephalon with triangular median lobe	***P.scaber***

## ﻿Discussion

*Porcellioaguerensis* Orihuela-Rivero, sp. nov. fits into the Atlantic group (“*scaber*”) as defined by Vandel (1951), sharing the male pleopod 1 exopod with a respiratory field located in a markedly lateral position and with a short posterior lobe. Also in common with the group, it has a “primitive” glandular system (plesiomorphic character state not yet tested by molecular analysis) associated with the margin of the epimera, in this case enclosed in a sector of a marginal groove. Within this group, the described species is quite characteristic for its somatic, integumentary and sexual characters. However, we can find similarities with other species, both continental and insular.

Among the continental species, the closest one is found in the Betic-Rifian subgroup as defined by Vandel (1958) (not to be confused with the Betic-Rifian or “*hoffmannseggii*” group). This group includes three species, *Porcelliodebueni* Dollfus, 1892, *Porcelliocolasi* Vandel, 1958, and *Porcelliohumberti* Paulian de Félice, 1939, exclusive to the Iberian Peninsula and Morocco.

*Porcelliohumberti*, which is native from Morocco and the southern Iberian Peninsula, shares a convex body with *P.aguerensis* Orihuela-Rivero, sp. nov., together with a glandular system with the same configuration, with the glandular pores confined to a region of a marginal groove; dorsal surface without gra­nules, with conspicuous noduli laterales; a poorly developed cephalic median lobe; and the male pleopod 1 exopod with similar characteristics (see Vandel 1958; Taiti and Rossano 2015). However, *P.aguerensis* Orihuela-Rivero, sp. nov. primarily differs from *P.humberti* in having: higher d/c coordinate values, with a peak on pereonite 4; the posterior margin of the first pereonites sinuous; telson with a distinctly rounded tip; and second antennae without sexual dimorphism. It should also be mentioned that, as Paulian de Félice (1939) commented with respect to *P.humberti*, *P.aguerensis* Orihuela-Rivero, sp. nov. has superficial similarities with *Porcelliogallicus* Dollfus, 1904; however, it differs in having a completely different glandular system and configuration of the male pleopod 1 exopod, which explains its inclusion in a different group (western Mediterranean or “*provincialis*” group) as proposed by Vandel (1956, 1962).

The other two species of the Betic-Rifian subgroup, *P.colasi* and *P.debueni*, are endemic to the Iberian Peninsula, and Vandel (1958) considers them slightly more derived forms than *P.humberti*. *Porcellioaguerensis* Orihuela-Rivero, sp. nov. shares some characteristics with both, such as a strongly convex body; smooth dorsal surface (although in the continental species there are flattened granules); poorly developed cephalic lobes; posterior margin of the first pereonites sinuous; telson with rounded tip, variable in *P.debueni* (see Vandel 1946, 1958); and male pereopod 7 ischium with concave ventral margin. In addition, with *P.colasi* it also shares a peak on d/c coordinates on pereonite 4. However, *P.aguerensis* Orihuela-Rivero, sp. nov. is easily differentiated from both species by the morphology of the male pleopod 1 exopod, with respiratory field inden­ted, and by the configuration of the glandular system (see Vandel 1946, 1958).

Similarities with continental taxa can also be found in other group of species formerly called “*dispar*” by Vandel (1946), later integrated into the Atlantic group (Vandel 1951, 1956, 1962). Among them, *Porcellioherminiensis* Vandel, 1946, from Iberian Peninsula, undoubtedly stands out, sharing the following with the new species: a convex body; glandular system with a similar configuration (see Vandel 1946); dorsal surface with conspicuous noduli laterales; poorly developed cephalic median lobe; and posterior margin of the first pereonites sinuous. *Porcellioaguerensis* Orihuela-Rivero, sp. nov. differs from this species by its completely smooth dorsal surface; its higher d/c coordinates; its telson with rounded tip and lateral margins without marked angles; the absence of antennal sexual dimorphism; the presence of pereopod 7 sexual dimorphism; and the male pleopod 1 exopod without such a developed tip.

In addition to the continental species mentioned above, there are also several Canary Islands species with which *P.aguerensis* Orihuela-Rivero, sp. nov. shares characteristics. Among these, *Porcelliocentralis* Vandel, 1954, endemic to the laurel forest of Gran Canaria island, shares a markedly convex body; a glandular system with the same configuration; similar d/c profile coordinates, although lower in *P.centralis* (Fig. 3C); poorly developed and rounded cephalic lobes; posterior margin of the first pereonites sinuous; and uropods of similar morphology with very oblique protopods. *Porcellioaguerensis* Orihuela-Rivero, sp. nov. differs from *P.centralis* mainly by its completely smooth dorsal surface (in contrast, *P.centralis* has granules, although not very apparent); by the morpho­logy of the posterior lobe of the male pleopod 1 exopod, which in *P.centralis* is totally truncated; and by the presence of sexual dimorphism in the pereopod 7.

*Porcellioaguerensis* Orihuela-Rivero, sp. nov. also shares similarities to *Porcelliomeridionalis*Vandel 1954, a species from the laurel forest of La Gomera and El Hierro: a glandular system with the same configuration; a poorly protru­ding cephalic median lobe; the posterior margin of the first pereonites sinuous; and a male pleopod 1 exopod with a short but individualized posterior lobe (although with different morphology). However, *P.aguerensis* Orihuela-Rivero, sp. nov. differs from this species by its smaller size (more than 10 mm in *P.meridionalis*); by its totally smooth dorsal surface, with higher d/c coordinates; the lateral cephalic lobes less developed and rounded; and by the pre­sence of sexual dimorphism in the pereopod 7.

On the island of Tenerife, *P.aguerensis* Orihuela-Rivero, sp. nov. also pre­sents similarities, although to a lesser extent, with *Porcelliomartini* Dalens, 1984 with which it has in common a convex body, the absence of granules and the configuration of the male pleopod 1 exopod. However, the glandular system configuration and the presence of sexual dimorphism in the pereopod 7 clearly distinguish it (see Dalens 1984). Moreover, the morphology of the male pleopod 1 exopod of the new species is similar to that of *Porcellioseptentrionalis* Vandel, 1954 and *Porcellioanagae*, but the rest of characteristics are very different.

In line with the suggestion of Vandel (1958), these morphological similarities argue for a possible relationship between the hypothetical most “primitive” continental species of *Porcellio* of the Atlantic group (“*scaber*”), represented by the Betic-Rifian subgroup, with some of the more “primitive” species of the Canary Islands, such as *Porcelliocentralis*, *Porcelliomeridionalis*, and *Porcellioaguerensis* Orihuela-Rivero, sp. nov. We also propose a close relationship among these three Canary Island species, which are typical of laurel forests. Both hypotheses can be verified in the future using molecular techniques.

Regarding its conservation status, there remains little understanding of the chorology of the Canary Islands woodlice so the extent to which *P.aguerensis* Orihuela-Rivero, sp. nov. is endemic to the laurel forest of the Anaga is unclear. However, we can assert that in the sampled area, the species would appear to be under intense pressure due to the presence of *Armadillidiumvulgare*, an invasive species associated to warmer environments at lower altitudes (Arndt and Mattern 2005). Our sampling at different altitudes indicates that it may be displacing native species, such as *Porcellioanagae*, towards higher altitudes (unpublished data), a phenomenon already studied on other islands by Arndt and Mattern (2005). This situation is especially alarming for already scarce species such as *Porcellioaguerensis* Orihuela-Rivero, sp. nov.

The new species is currently known only from the protected natural area of Anaga Rural Park, so habitat destruction does not pose a threat. However, being in an area highly frequented by visitors, a thorough assessment is recommended.

## ﻿Conclusion

This study increases the species richness of isopods in the Canary Islands to a total of 36 native species, 30 which are considered endemic. The morpho­logical characteristics of the new species described, *Porcellioaguerensis* Orihuela-Rivero, sp. nov., support the connections between the continental and insular fauna established by previous authors. In the same way, a possible process of radiation between endemic laurel forest species of the archipelago are evidenced. Future studies based on molecular techniques are expected to test these hypotheses.

## Supplementary Material

XML Treatment for Porcellio aguerensis

## Footnotes

The validity of Porcellio septentrionalis has been questioned by Hoese (1984, 1985). We hope that further comprehensive studies will solve this issue.

## ﻿Additional information

### Author ORCIDs

Raúl Orihuela-Rivero https://orcid.org/0000-0001-6682-0106

Carmen Balibrea https://orcid.org/0000-0002-3143-9329

Víctor Noguerales https://orcid.org/0000-0003-3185-778X

Heriberto López https://orcid.org/0000-0001-6988-5204

Pedro Oromí https://orcid.org/0000-0003-4855-5542

### Data availability

All of the data that support the findings of this study are available in the main text.
